# Implementation of a crash cesarean section policy and its impact on maternal and neonatal outcomes at King Abdulaziz University Hospital: A retrospective study

**DOI:** 10.1097/MD.0000000000040645

**Published:** 2024-11-29

**Authors:** Ebtihal Alhawsawi, Nedaa Bahkali, Sarah Aljadani, Abdulaziz Jambi, Alhanouf Almwled, Samera Al Basri

**Affiliations:** a Department of Obstetrics and Gynecology, Faculty of Medicine, King Abdulaziz University, Jeddah, Kingdom of Saudi Arabia; b Faculty of Medicine, King Abdulaziz University, Jeddah, Kingdom of Saudi Arabia; c Department of Obstetrics and Gynecology, East Jeddah hospital, Jeddah, Kingdom of Saudi Arabia.

**Keywords:** crash cesarean section, decision-to-delivery time, gestational age, maternal outcome, neonatal outcome

## Abstract

A typical surgical technique for pregnant women with potentially fatal problems affecting the mother or fetus is an emergency cesarean section (ECS). The decision-to-delivery interval (DDI) for ECS should be within 30 minutes. The objective of this study was to investigate crash ECS indications and effects on maternal and neonatal outcomes. In this retrospective study, all women undergoing crash cesarean section (CS) at Obstetrics and Gynecology department at King Abdulaziz University hospital, Jeddah, Saudi Arabia during 2022 and 2023 were evaluated. Data about demographic and obstetric characteristics of mother, ECS indications, DDI, and outcomes for mothers and newborns was gathered from the patient’s sheet. One hundred 3 crash CS were performed during study period. Crash CS indications were fetal bradycardia (64.1%), prolapsed cord (24.3%), uterine rupture (6.8%), and severe antepartum hemorrhage (4.9%). D-D time range from 2 to 30 minutes. DDI was ≤15 minutes in 90 patients (87.4%) and >15 minutes in 13 patients (12.6%). Gestational age was <32 weeks (16.7%), between 32 and <37 weeks (15.5%), and ≥37 weeks (68.0%). Good maternal outcome was reported in 89 (86.4%), while 24 (13.6%) had complications. Intensive care unit maternal admission was significantly higher in gestational age 32 to <37 weeks versus <32 weeks and ≥37 weeks of gestation (18.8% vs 5.9% and 2.9%, *P* = .050). Fetal outcome was good in 69 (67.0%), while 34 (33.0%) had complications. Neonatal body weights, Apgar score at 1, 5, 10 minutes, and umbilical cord arterial pH were significantly decreased in preterm versus termed neonates (*P* < .0001, *P* < .0001, *P* < .0001, *P* = .014, and *P* = .003). Moreover, respiratory distress syndrome, jaundice, intubation, neonatal deaths, and sepsis were significantly higher in preterm versus term deliveries (*P* < .0001, *P* = .029, *P* < .0001, *P* = .010, and *P* = .031). Good neonatal outcome was significantly higher (*P* < .0001); while respiratory distress syndrome was significantly lower (*P* = .007) in deliveries with DDI ≤ 15 minutes versus > 15 minutes. The 30-minute standard for DDI time interval may be a feasible guideline at least for level-3 hospitals. Crash CS indication was mostly due to fetal bradycardia. The maternal and neonatal outcomes were better in term than preterm deliveries. The positive effect of very short intervals on neonatal outcome still needs to be proven.

## 1. Introduction

Cesarean section (CS) is the most common major procedure in obstetrics. It is a difficult, interdisciplinary surgery that poses hazards for both the mother and the newborn. Emergency CS (ECS) or crash CS is performed when the mother’s or the fetus’s life is in imminent danger.^[1]^ About 1% of pregnancies require an ECS.^[1]^ Decision-to-delivery interval (DDI) refers to the interval between deciding to provide emergency medical services and the neonate’s delivery^[2]^ covering the time needed to prepare the patient and the operating room (OR), administer the anesthesia, and the procedure from skin incision to delivery.^[3]^ At least 7 experts are required for the procedure: a midwife, a theater nurse to help with the surgery, an obstetrician and an assistant, an anesthetist and a skilled assistant, and a pediatrician to take the infant. The staff must be assembled before beginning the complicated procedure.^[4]^ The ideal DDI, according to the American College of Obstetricians and Gynecologists (ACOG) and the Royal College of Obstetricians and Gynecologists, is under 30 minutes,^[2,5]^ while it is ≤ 20 minutes in Germany.^[6]^ The American Academy of Pediatrics and ACOG list uterine rupture, placental abruption, bleeding from placenta previa, and umbilical cord prolapse as examples of conditions that might require an ECS.^[7]^ Despite the paucity of available evidence, the 30-minute reaction time is recommended. However, there is no proof that the 30-minute guideline enhances the mother or fetus outcomes.^[8]^ A 30-minute DDI is also challenging in many clinical practice contexts due to various issues.^[9–11]^ DDI could be shortened by planning ahead, placing the OR next to the delivery room, having access to obstetricians and anesthesiologists, and fostering productive cooperation. Between 40% and 65% of fetal distress instances meet the 30-minute objective in developed countries,^[10–13]^ while the rates are 0% to 20% in developing countries.^[14–16]^ This means that the access of laboring women to professional treatment in developing countries should be improved.

This study aimed to assess the effect of a crash CS policy on fetal and maternal outcomes and determine whether it is appropriate to be implemented in all Saudi Arabian centers. We also assessed the impact of gestational age (GA) and DDI duration on maternal and neonatal outcomes.

## 2. Patients and methods

The institutional review board of King AbdulAziz University has approved this study. The present retrospective study was conducted at the Obstetrics and Gynecology Department, King Abdulaziz University Hospital, a tertiary care hospital in Jeddah, Saudi Arabia included 103 patients who underwent crash CS between January 2022 and December 2023. Our hospital’s crash CS protocol, created in 2021, outlines the logistical steps for all Obstetrics and Gynecology Department doctors and nursing staff, midwives, emergency and OR nursing staff and technicians, anesthetists, pediatricians, blood transfusion service, and switchboard. The following conditions were classified as requiring crash CSs: sustained fetal bradycardia, umbilical cord prolapse, major antepartum hemorrhage (APH), maternal and fetal hemodynamic instability or shock (including vasa previa, placental abruption, and placenta previa), uterine rupture, and perimortem deliveries. A senior doctor should take the decision on crash CS, and the announcement can be made by any available healthcare provider who knows the steps and information that should be provided to the switchboard on the unified number. This person should provide the code name and crash CS location (main OR or labor room [OR], whichever is closer to the patient). After announcing the code, the doctors and nurses must auscultate the fetal heart rate by Doppler, explain the situation to the patient, obtain verbal consent for crash CS, and transfer the patient immediately to the OR. Blood samples should be sent for group and save serum, cross-matching, complete blood count, liver function test, urea & electrolyte, and coagulation profile. The surgeon should scrub immediately. The OR charge nurse assigns a nurse in the holding area to bring prophylactic antibiotics (2 g cefazolin intravenous stat). The OR nurses should remove any metallic artifacts from the patient, insert a Foley catheter, and complete the OR gowning. The OR charge nurse assigns a nurse to retrieve blood products from the blood bank if needed. Upon the patient’s arrival to the OR, the anesthesia team, anticipating general anesthesia, prepares the appropriate drugs and equipment and obtains verbal consent from the patient for anesthesia. If the patient arrives at the OR without blood test results, the anesthetist should obtain the blood works and administer prophylactic antibiotics. The preferred number of attendees inside the OR is 12, including 3 in the anesthesia team (a doctor and 2 technicians), 3 doctors in the obstetric team, 3 in the pediatric team a doctor and 2 from neonatal intensive care unit (NICU) nurses, a midwife in the labor and delivery (L&D) team, and 2 OR nurses (1 for scrub and 1 for circulating). After surgery, the obstetricians complete the documentation, sign the patient’s consent form, complete the OR booking, prescribe needed medications, and write the patient note, including the indication for crash CS. The nurses calculate the interval in minutes between the decision to perform an ECS and the skin incision time. Subpar sterile conditions are used during surgery (bladder drainage, wide-spectrum prophylactic antibiotics, and fast abdomen disinfection). The Pfannenstiel skin incision and the transverse lower uterine segment incision were typically used, except in extremely preterm infants, where a vertical myometrial incision in the uterine body was made. All patients were treated during labor following the established clinical procedure in our hospital. The dataset retrieved from the medical records included the patient’s demographic and obstetric characteristics, indications of crash CS, and maternal and neonatal outcomes. The recorded maternal complications were intensive care unit (ICU) admission, postpartum stay longer than 4 days, blood transfusion, surgical complications, pelvic collection, wound issues (for example, seroma, hematoma, and surgical site infection [SSI]), and maternal fatality. SSI diagnosis required incision erythema and purulent discharge. Neonatal complications such as respiratory distress syndrome (RDS), jaundice, congenital diseases and anomalies, intubation, placement on continuous positive airway pressure, hypoxic-ischemic encephalopathy, seizures, sepsis, necrotizing enterocolitis, asphyxia, nonimmune hydrops, do-not-resuscitate, stillbirth, and neonatal death were recorded. Neonatal weight, 1-, 5-, and 10-minute Apgar scores, umbilical cord arterial pH (UApH), prompt neonatal care, and newborn admission to the infant special care unit were also recorded. Comprehensive data about the clinical trajectory of every newborn hospitalized in a neonatal critical care unit was gathered, the study included all crash CSs cases with singleton pregnancy, the cases with multiple gestations or missing data were excluded from the study.

### 2.1. Statistical analysis

Continuous data are expressed as means ± standard deviations, parametric data as minimum and maximum, and categorized data as frequencies (%). Values were analyzed using IBM SPSS Statistics for Windows, Version 22.0 (IBM Corp., Armonk, NY). Normal data distribution was assessed using the Shapiro–Wilk test. One-way ANOVA and Tukey post hoc test was utilized to examine the data. Independent samples *t* test compared groups with normally distributed parametric data, and the Mann–Whitney *U* test and Kruskal–Wallis test compared abnormally distributed parametric data. Pearson Chi-squared test compared categorized data. Associations were assessed by Spearman correlation coefficients. Statistical significance was set at *P* < .05.

## 3. Results

Our hospital recorded 4918 deliveries during 2022 and 2023, 2561 in 2022, and 2357 in 2023. Crash and non-crash CSs totaled 1151, 565 in 2022 and 586 in 2023. Crash CS was performed in 103 deliveries equaling 2.1% of all ECS. Most of the patients involved were Saudi (65.0%). The maternal age was 16 to 45 (30.99) years; gravidity was 1 to 8 (3.14); parity was 0 to 6 (1.76). GA was 32 to 41 (36.3) months. More patients were at a GA ≥ 37 weeks (68.0%) than < 32 weeks (16.5%) or between 32 and 37 weeks (15.5%). One or more previous laparotomies were performed in 31.1% of the patients. The coding site was L&D in 64.1% of the cases, and most (70.9%) were admitted to the L&D OR (Table 1).

**Table 1:** Demographic and obstetrics characteristics of patients.

Characteristics	All patients (n = 103)
Nationality	
Saudi	67 (65.0%)
Non-Saudi	36 (35.0%)
Maternal age (years)	30.99 ± 6.26 (16–45)
Gravida	3.14 ± 2.04 (1–8)
Para	1.76 ± 1.74 (0–6)
Gestational age (GA) (months)	36.30 ± 4.61 (23–41)
GA < 32 week	17 (16.5%)
GA 32 to <37 weeks	16 (15.5%)
GA ≥ 37 weeks	70 (68.0%)
Surgical history	
No previous surgery	71 (68.9%)
Previous 1 or more laparotomy	32 (31.1%)
Site of coding	
L&D	66 (64.1%)
ER	30 (29.1%)
OB ward	7 (6.8%)
To which OR	
Main OR	30 (29.1%)
L&D OR	73 (70.9%)

GA = gestational age, L&D = labor and delivery, OR = operating room.

Indications for ECS, DDI are presented in (Table 2.) The main indication for ECS was fetal bradycardia (n = 66, 64.1%), followed by cord prolapse (n = 25, 24.3%), uterine rupture (n = 7, 6.8%), and severe APH (n = 5, 4.9%). All ECS were performed within 30 minutes. The DDI was 10.44 ± 4.45 (2–30) min. The DDI was ≤ 15 minutes in 90 patients (87.4%) and > 15 minutes in 13 (12.6%). A good maternal outcome was reported in 89 cases (86.4%), while maternal complications were reported in 24 patients (13.6%), and included ICU admission (5.8%), postpartum stay > 4 days (3.9%), blood transfusion (2.9%), SSI (2.9%), surgical complications (1.9%), and pelvic collection (1.0%). Maternal death was reported in only 1 case (1.0%). Maternal ICU admission in gestational weeks 32 to 37 was significantly higher than in weeks < 32 and ≥ 37 (18.8% vs 5.9% and 2.9%, *P* = .050). The neonatal outcomes (n = 103) are listed in (Table 3).

**Table 2:** Indication of surgery, time from announcement to baby out, and maternal outcome of crash cesarean section according to gestational age (GA).

Parameters	All patients (n = 103)	GA < 32 week (n = 17, 16.5%)	GA 32 to <37 weeks (n = 16, 15.5%)	GA ≥ 37 weeks (n = 70, 68.0%)	Significance
Primary indication of the surgery					*P* = .120
Fetal bradycardia	66 (64.1%)	12 (70.6%)	9 (56.2%)	45 (64.3%)	
Cord prolapses	25 (24.3%)	4 (23.5%)	3 (18.8%)	18 (25.7%)	
Uterine rupture	7 (6.8%)	-	1 (6.2%)	6 (8.6%)	
Sever APH	5 (4.9%)	1 (5.9%)	3 (18.8%)	1 (1.4%)	
Decision-to-delivery interval (DDI) (min)	10.44 ± 4.45 (2–30)	10.76 ± 4.82 (5–19)	11.16 ± 6.20 (2–30)	10.20 ± 3.93 (4–22)	*P* = .719
≤15 minutes	90 (87.4%)	13 (76.5%)	14 (87.5%)	63 (90.0%)	*P* = .321
>15 minutes	13 (12.6%)	4 (23.5%)	2 (12.5%)	7 (10.0%)	
Maternal outcome					
Good	89 (86.4%)	15 (88.2%)	12 (75.0%)	62 (88.6%)	*P* = .350
ICU admission	6 (5.8%)	1 (5.9%)	3 (18.8%)	2 (2.9%)	***P* = .050**
Postpartum stay more than 4 days	4 (3.9%)	1 (5.9%)	1 (6.2%)	2 (2.9%)	*P* = .734
Blood transfusion	3 (2.9%)	–	2 (12.5%)	1 (1.4%)	*P* = .101
Surgical site infection (SSI)	3 (2.9%)	1 (5.9%)	–	2 (2.9%)	*P* = .603
Surgical complications (injury to nearby organs)	2 (1.9%)	–	1 (6.2%)	1 (1.4%)	*P* = .369
Pelvic collection	1 (1.0%)	–	–	1 (1.4%)	*P* = .678
Maternal death	1 (1.0%)	1 (5.9%)	–	–	*P* = .078

Bold value indicates significant difference between group.

**Table 3:** Neonatal outcomes of crash cesarean section in preterm and term neonates.

Outcomes	All patients (n = 103)	GA < 37 (n = 33)	GA ≥ 37 (n = 70)	Significance
Neonatal admission				***P* < .0001**
Nursery	64 (62.1%)	6 (18.2%)	58 (82.9%)	
NICU	35 (34.0%)	24 (72.7%)	11 (15.7%)	
Neonatal deaths (ND)	3 (2.9%)	3 (9.1%)	–	
Still birth	1 (1.0%)	–	1 (1.4%)	
Neonatal weights (kg)	2.32 ± 0.84 (0.68–4.86)	1.79 ± 0.75 (0.68–3.40)	3.00 ± 0.56 (1.97–4.86)	***P* < .0001**
Apgar 1 minute	5.22 ± 2.91 (0–9)	3.33 ± 2.72 (0–8)	6.11 ± 2.56 (0–9)	***P* < .0001**
Apgar 5 minutes	7.58 ± 2.83 (0–10)	5.73 ± 3.26 (0–9)	8.46 ± 2.11 (0–10)	***P* < .0001**
Apgar 10 minutes	6.14 ± 2.89 (0–10)	4.73 ± 3.04 (0–8)	7.70 ± 1.77 (4–10)	***P* = .014**
Umbilical cord arterial pH	7.14 ± 0.20 (6.60–7.50)	7.05 ± 0.25 (6.60–7.50)	7.18 ± 0.15 (6.60–7.35)	***P* = .003**
<7.10	24 (25.5%)	12 (38.7%)	12 (19.0%)	*P* = **.037**
≥7.10	70 (74.5%)	19 (61.3%)	51 (81.0%)	
Neonatal outcomes				
Good	69 (67.0%)	10 (30.3%)	59 (84.3%)	***P* < .0001**
Respiratory distress syndrome (RDS)	12 (11.7%)	10 (30.3%)	2 (2.9%)	***P* < .0001**
Jaundice	9 (8.7%)	6 (18.2%)	3 (4.3%)	***P* = .029**
Congenital diseases or anomalies	8 (7.8%)	4 (12.1%)	4 (5.7%)	*P* = .225
Intubated	7 (6.8%)	7 (21.2%)	–	***P* < .0001**
Hypoxia ischemic encephalopathy (HIE)	7 (6.8%)	4 (12.1%)	3 (4.3%)	*P* = .146
On CPAP	4 (3.9%)	3 (9.1%)	1 (1.4%)	*P* = .096
Seizure	3 (2.9%)	-	3 (4.3%)	*P* = .310
Sepsis	3 (2.9%)	3 (9.1%)	-	***P* = .031**
Necrotizing enterocolitis (NEC)	2 (1.9%)	2 (6.1%)	-	*P* = .101
Asphyxia	1 (1.0%)	–	1 (1.4%)	*P* = .680
Nonimmune hydrops	1 (1.0%)	1 (3.0%)	–	*P* = .320
Do-not-resuscitate (DNR)	1 (1.0%)	–	1 (1.4%)	*P* = .680
Still birth	3 (2.9%)	1 (3.0%)	2 (2.9%)	*P* = .690
Neonatal deaths (NND)	3 (2.9%)	3 (9.1%)	–	***P* = .010**

Bold values indicate significant difference between group.

**Figure 1.**
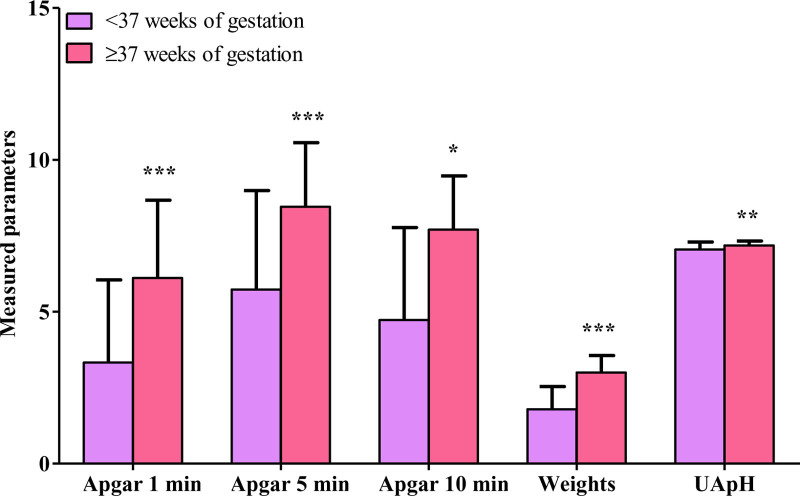
Apgar scores after 1, 5, and 10 minutes, neonatal weights (kg) and umbilical arterial pH (UApH) of the neonates delivered by emergency cesarean section in preterm and term neonates. The differences between the gestational age (GA) groups were significant * *P* < .050, ** *P* < .010, and *** *P* < .0001.

Neonates were admitted to the nursey (62.1%) or NICU (34.0%), while 3 (2.3%) neonates died and 1 (1.0%) was stillbirth. Neonatal weight was 0.68 to 4.86 (2.32) kg. The mean 1-, 5-, and 10-minute Apgar scores were 5.22, 7.58, and 6.14. The UApH was 6.60 to 7.50 (7.14). The outcome was good in 69 neonates (67.0%), while 34 (33.0%) reported complications, including RDS (11.7%), jaundice (8.7%), congenital diseases or anomalies (7.8%), intubation (6.8%), hypoxic-ischemic encephalopathy (6.8%), placement on continuous positive airway pressure (3.9%), stillbirth (n = 3, 2.9%), neonatal deaths (n = 3, 2.9%), seizure (n = 3, 2.9%), sepsis (n = 3, 2.9%), necrotizing enterocolitis (n = 2, 1.9%), asphyxia (n = 1, 1.0%), nonimmune hydrops (n = 1, 1.0%), and do-not-resuscitate (n = 1, 1.0%). Because of the high incidence of crash CS in premature deliveries, neonatal outcomes were compared between pregnancies at GA of < 37 and ≥ 37 weeks. Preterm and term neonates differed in the admission unit (*P* < .001). Most preterm neonates were admitted to the NICU (72.7%), while term neonates were mostly admitted to the nursery (82.9%). Body weight, 1-, 5-, and 10-minute Apgar scores, and UApH in preterm neonates were significantly lower than in term neonates (*P* < .001, *P* < .001, *P* < .001, *P* = .014, and *P* = .003; Fig. 1).

The rates of good outcomes (84.3% vs 30.3%, *P* < .001) and UApH > 7.10 (81.0% vs 61.3%, *P* = .037) in term neonates were significantly higher than in preterm neonates. Conversely, the rates of RDS, jaundice, intubation, neonatal deaths, and sepsis were significantly higher in preterm deliveries than term deliveries (*P* < .001, *P* = .029, *P* < .001, *P* = .010, and *P* = .031).The good outcome rate in neonates with a decision-to-incision interval of ≤ 15 minutes was higher than in those with an interval of > 15 minutes (70.0% vs 46.2%, *P* < .001), while the RDS rate was significantly lower (7.8% vs 38.5%, *P* = .007; Table 4).

**Table 4:** Neonatal outcomes of crash cesarean section according to decision-to-delivery interval (DDI).

Outcomes	≤15 minutes (n = 90)	>15 minutes (n = 13)	Significance
Neonatal admission			*P* = .135
Nursery	59 (65.6%)	5 (38.5%)	
NICU	27 (30.0%)	8 (61.3%)	
Still birth	1 (1.1%)	–	
Neonatal deaths	3 (3.3%)	–	
Neonatal weights (kg)	2.67 ± 0.77 (0.79–4.59)	2.25 ± 1.18 (0.68–4.86)	*P* = .089
Apgar 1 minute	3.22 ± 2.93 (0–9)	5.23 ± 2.89 (0–9)	*P* = .948
Apgar 5 minutes	7.54 ± 2.92 (0–10)	7.85 ± 2.19 (3–10)	*P* = .988
Apgar 10 minutes	5.89 ± 3.03 (0–10)	7.67 ± 1.15 (7–9)	*P* = .415
Umbilical cord arterial pH	7.13 ± 0.19 (6.60–7.35)	7.15 ± 0.25 (6.60–7.50)	*P* = .766
<7.10	21 (25.6%)	3 (25.0%)	0.636
≥7.10	61 (74.4%)	9 (75.0%)	
Fetal outcomes			
Good	63 (70.0%)	6 (46.2%)	***P* < .0001**
RDS	7 (7.8%)	5 (38.5%)	***P* = .007**
Still birth	3 (3.3%)	–	*P* = .654
Neonatal deaths	2 (2.2%)	1 (7.7%)	*P* = .336
Intubated	5 (5.6%)	2 (15.4%)	*P* = .214
Jaundice	7 (7.8%)	2 (15.4%)	*P* = .317
Congenital diseases or anomalies	8 (8.9%)	–	*P* = .326
Sepsis	2 (2.2%)	1 (7.7%)	*P* = .336
On CPAP	3 (3.3%)	1 (7.7%)	*P* = .422
Asphyxia	–	1 (7.7%)	*P* = .126
Hypoxia ischemic encephalopathy	6 (6.7%)	1 (7.7%)	*P* = .623
Seizure	2 (2.2%)	1 (7.7%)	*P* = .336
NEC	2 (2.2%)	–	*P* = .762
Nonimmune hydrops	1 (1.1%)	–	*P* = .974
DNR	1 (1.1%)	–	*P* = .974

Bold values indicate significant difference between group.

CPAP = continuous positive airway pressure, DNR = do-not-resuscitate, NEC = necrotizing enterocolitis, NICU = neonatal intensive care unit, RDS = respiratory distress syndrome.

**Figure 2.**
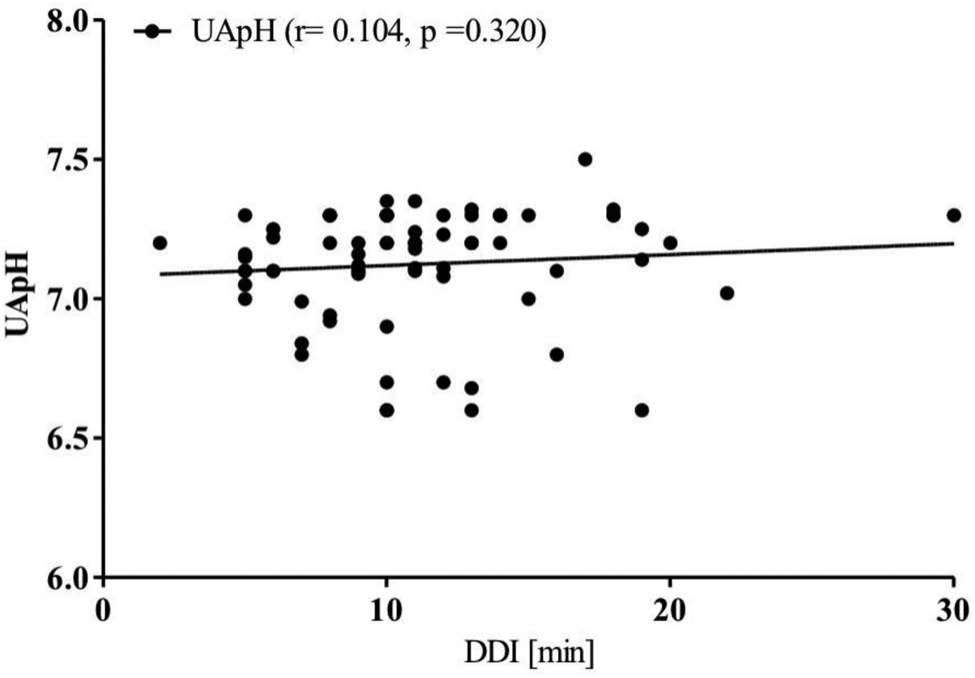
Correlation between umbilical cord arterial PH (UApH) and decision-to-delivery interval (DDI).

The correlation between the UApH and DDI was insignificant (*R* = 0.104, *P* = .320; Fig. 2).

## 4. Discussion

The American Academy of Pediatrics and ACOG states that “every institution offering an obstetric service should have the capability of responding to obstetric emergencies.” hospitals should be able to start a CS within the widely agreed 30 minutes of the decision. Establishing this as a capacity standard rather than an absolute necessity. Obstetric malpractice claims are frequently viewed as being unjustifiable if a cesarean birth was not performed within 30 minutes.^[17]^ This implies the 30-minute timeframe is a prerequisite based on the assumption that delivering the baby within 30 minutes or less will result in a positive outcome.^[18]^ In 2022 to 2023, our tertiary hospital performed 103 crash CS within 30-minute, accounting for 2.09% of deliveries around 60% of 446 ECSs were performed within 30 minutes.^[19]^ Three further trials examined the outcomes of 692 women and their infants in connection to the DDI for ECS.^[20]^ concluded that an ECS with a DDI > 30 minutes did not necessarily negatively impact on the newborn’s outcomes. DDIs greatly vary by hospital level, the median might exceed 69 minutes in level-1/2 hospitals.^[20]^ A precise urgency definition is needed to evaluate if 30-minutes is a viable audit standard. The primary indication for a crash CS was fetal bradycardia (64.1%), cord prolapse (24.3%), uterine rupture (6.8%), and severe APH (4.9%). Hillemanns et al reported irregular fetal heart rate as the most common reason for ECS.^[21]^ Bloom et al^[12]^ reported that 62% of cesarean deliveries for non-reassuring fetal heart rate and 98% for obstetric accidents defined as umbilical cord prolapse, placental abruption or previa, or uterine rupture met the 30-minute guideline. All crash CS in this study were performed within ≤ 30 minutes, with a DDI of ≤ 15 minutes in (87.4%) and > 15 minutes in (12.6%). Damtew^[22]^ observed a decision interval of 30 minutes in 21.9% of ECS. Hillemanns et al^[23]^ showed that all 75 ECS performed in 1987 to 1994 could be completed in <30 minutes, with a mean of 12.7 minutes. Singh et al^[24]^ emphasized the challenge of achieving the suggested DDI of ≤ 30 minutes. In their research, only 19% of the ECS procedures achieved a DDI of < 30 minutes. Khemworapong et al^[4]^ reported that only 3.5% of the ECS procedures had a DDI of < 30 minutes, while the median was 82 minutes. Katz et al^[25]^ expert recommend interventions within < 5 minutes for maternal cardiac arrest as quick action may also benefit some ECS, but DDI frequently exceeds 30 minutes in developing countries.^[9–11,13–16]^ However the development and implementation of a standard algorithm to speed up the time to incision for unplanned, ECS, significantly reduced the time to the decision to perform an incision.^[26]^ This study reported good maternal outcomes in 89 patients (86.4%) and maternal complications in 24 (13.6%), including ICU admission (5.8%), postpartum stay for > 4 days (3.9%), blood transfusion (2.9%), SSI (2.9%), surgical complications (1.9%), and pelvic collection (1.0%). Maternal death was reported in 1 case (1.0%). 17 (16.5%) had GA < 32 weeks, 16 (15.5%) had GA of 32 to 37 weeks, and 70 (68.0%) had GA of ≥ 37 weeks. Maternal ICU admission was higher when the GA was 32 to 37 weeks than < 32 or ≥ 37 weeks (18.8% vs 5.9% and 2.9). Moroz et al^[27]^ reported that mothers with repeated ECS had a higher risk of complications if the DDI was ≤ 2 minutes. However, other research indicated that the length of DDI did not affect the mother’s health.^[12,28]^ Hillemanns et al^[21]^ ECS had higher blood loss and transfusion rates than controls, but this difference was not significant after adjusting for placental issues. The 1 maternal death was likely due to preexisting conditions and COVID-19. Low SSI (2.9%) and pelvic collection (1.0%) rates were likely due to perioperative antibiotics.^[29,30]^ In this study, most neonates were admitted to the nursery (62.1%), followed by NICU (34.0%). Three neonatal deaths (2.9%) and 1 stillbirth (1.0%) were reported. Good outcomes were reported (67.0%), while (33.0%) had complications. Preterm neonates were more likely to be admitted to NICU (72.7%) and had poorer outcomes than term neonates. UApH, Apgar scores, and neonatal weight were lower in preterm infants. Bloom et al^[12]^ neonates delivered within 30 minutes were more likely to have acidemia and intubation, though 2 died from asphyxia. Infants born via emergency cesarean had poorer short-term outcomes, but gestational age and underlying conditions are the main determinants of prognosis.^[21]^ The newborns in our ECS cohort were depressed, as evidenced by their UApH and Apgar scores. A UApH under 7.10 was found in about 25.5% of newborns, within the range of previous research.^[31]^ Evidently, we successfully lessened the percentage of depressed newborns in our series through short DDIs. The rate of extremely preterm newborns, which was significantly greater than in other studies, may have contributed to the high rate of depressed infants because low birth weight was major predictors of adverse neonatal outcomes during ECS.^[32]^ Critical obstetric complications such as cord prolapse, placenta previa hemorrhage, and placental abruption were linked in most instances to extremely preterm gestations of under 32 weeks. The 1-, 5-, and 10-minute Apgar scores and UApH in our sample were lower in the preterm than in term deliveries. Berlit et al^[33]^ and Brandt et al^[6]^ showed that GA protects against poor neonatal outcomes. However, preterm morbidities that result in falsely low Apgar scores need to be considered as a potential confounding factor when analyzing these data.^[34]^ According to Catlin et al,^[35]^ low Apgar scores are frequently seen in normal preterm infants, characterized as having normal UApH and base excess. However, this might be due to physiological developmental immaturity rather than fetal distress. In this study, good neonatal outcomes were significantly higher (70.0% vs 46.2%), while RDS was significantly lower (7.8% vs 38.5%) in those with DDI ≤ 15 minutes than in those with DDI > 15 minutes. Schauberger et al^[36]^ and Roemer et al^[37]^ revealed that a notably higher proportion of newborns in the short DDI group had low 5-minute Apgar scores than those with longer DDI. This might be due to 2 factors. It is possible that less urgent steps were taken to handle less serious circumstances. However, it is also possible that the outcome was not as expected and that the disease improved independently due to a slower reaction. At those crucial moments, prompt action, despite all the drawbacks of a rushed procedure, is still recommended to lessen the risk that the newborn would sustain more harm. The findings of Bloom et al^[12]^ indicate that obstetricians can usually prioritize ECS deliveries appropriately when they have the means to start the procedure in <30 minutes, which is probably the intention behind the released guidelines. However, it also appears that delivering a baby within 30 minutes does not in and of itself ensure their safety. Here is the paragraph in a more concise form: about 25.5% of neonates had UApH < 7.10, which was likely due to the high rate of extreme prematurity. Good outcomes were higher (70.0% vs 46.2%) and RDS lower (7.8% vs 38.5%) with DDI ≤ 15 minutes versus > 15 minutes. However, prompt action is still recommended even with drawbacks of rushed procedures, as delivery within 30 minutes does not guarantee safety.

### 4.1. Strength and limitation

This large cohort study was conducted at a tertiary care hospital with a consistent policy and expertise in data collection on drug–drug interactions, cord blood sampling, and Apgar scoring, ensuring data quality and reducing bias. However, generalization to other healthcare settings may be limited as it lacked a control group. The retrospective design and missing historical data prior to the established ECS protocol are further limitations. Despite these constraints, this work contributes to the limited knowledge on ECS.

## 5. Conclusion

The primary crash CS indication was fetal bradycardia. Maternal and neonatal outcomes were better in term than preterm deliveries. The positive effect of very short intervals on neonatal outcomes still needs to be proven by large multicenter prospective studies. We highly recommend implementing crash CS policy in other hospitals to reduce DDI time, organize the process, reduce the burden on healthcare providers and facilitate communication between them in times of emergency.

## Author contributions

**Data curation:** Ebtihal Alhawsawi, Abdulaziz Jambi, Alhanouf Almwled.

**Formal analysis:** Sarah Aljadani.

**Methodology:** Nedaa Bahkali.

**Project administration:** Ebtihal Alhawsawi, Nedaa Mohammed Bahkali.

**Validation:** Samera Al Basri.

**Writing – original draft:** Ebtihal Alhawsawi, Sarah Aljadani, Abdulaziz Jambi, Alhanouf Almwled.

**Writing – review & editing:** Ebtihal Alhawsawi, Nedaa Bahkali, Samera Al Basri.
